# The scaffold protein IQGAP1 is crucial for extravasation and metastasis

**DOI:** 10.1038/s41598-020-59438-w

**Published:** 2020-02-12

**Authors:** Jess D. Hebert, Chenxi Tian, John M. Lamar, Steffen Rickelt, Genevieve Abbruzzese, Xiaotie Liu, Richard O. Hynes

**Affiliations:** 1 Department of Biology, Massachusetts Institute of Technology, Cambridge, Massachusetts 02139 USA; 2 Koch Institute for Integrated Cancer Research, Massachusetts Institute of Technology, Cambridge, Massachusetts 02139 USA; 3 Department of Molecular and Cellular Physiology, Albany Medical College, Albany, New York 12208 USA; 4 Howard Hughes Medical Institute, Chevy Chase, Maryland 20815 USA

**Keywords:** Breast cancer, Metastasis, Melanoma, Mechanisms of disease

## Abstract

IQGAP1 is a scaffold protein involved in a range of cellular activities, including migration, invasion, adhesion and proliferation. It is also oncogenic in a variety of cancers, promoting primary tumor growth and invasiveness. However, the role of IQGAP1 in tumor progression and metastasis remains unclear. In this study, we use both knockdown and knockout of IQGAP1 to investigate its role in the metastatic cascade of both melanoma and breast cancer cells *in vivo*. We find that reduction of IQGAP1 expression decreases the formation of both spontaneous and experimental metastases, without limiting primary or metastatic tumor growth. Furthermore, IQGAP1 knockout significantly inhibits extravasation of tumor cells from circulation, possibly involving invadopodial function. By expressing mutant forms of IQGAP1 in a knockout context, we also determine that IQGAP1’s pro-metastatic functions are dependent on multiple domains and functions. These data demonstrate that IQGAP1 is crucial for metastasis *in vivo* through regulation of extravasation and suggest that it may represent a valid therapeutic target for inhibiting metastasis.

## Introduction

Metastasis, the spread of a cancer from its original site to additional, secondary sites throughout the body, is the cause of the vast majority of cancer-related deaths^1^, and patient five-year survival rates drop precipitously once a cancer has metastasized^2^. Thus, there is a dramatic, unmet demand for treatments that can either eliminate metastatic cancer or prevent a tumor from spreading in the first place. However, current knowledge of the metastatic process is incomplete, and so previous anti-metastasis therapies have had very limited efficacy^3^. Consequently, there is a need to understand better the proteins that drive metastatic cancer, both to inform the design of future therapies as well as to identify additional therapeutic targets.

The scaffold protein IQGAP1 has been an active subject of investigation for its oncogenic potential due to its involvement in multiple functions classically associated with cancer, including proliferation, migration, invasion and cell-cell adhesion^4^. Indeed, IQGAP1 is elevated at both the mRNA and protein level in a variety of cancers, and its levels correlate with aggressiveness^4^. Overexpression of IQGAP1 promotes growth and invasiveness of xenograft tumors of MCF-7 breast cancer cells *in vivo*^5^, while IQGAP1 knockout mice are resistant to Ras-driven tumorigenesis^6^. The IQGAP family also contains two other proteins, IQGAP2 and IQGAP3, but IQGAP2 has been shown to have tumor-suppressive properties, while IQGAP3 has not been well studied^4^.

Direct evidence for the involvement of IQGAP1 itself in metastasis has been missing. We previously demonstrated that IQGAP1 is significantly upregulated in *in-vivo*-selected, highly metastatic melanoma tumor cells when compared with their parental counterparts^7^. Aside from this correlative evidence, further investigation of IQGAP1’s possible roles in metastasis has largely been limited to *in vitro* studies. Manipulation of IQGAP1 levels has been shown to affect the proliferation, migration and invasion of esophageal squamous cell carcinoma^8^, hepatocellular carcinoma^9^, glioma^10^ and breast cancer^5,11^ cells. Moreover, knockdown of IQGAP1 in a xenograft model of esophageal squamous cell carcinoma reduced primary tumor growth, with unclear effects on metastasis^8^. Accordingly, these data have thus far left unanswered the question of whether IQGAP1 truly regulates metastasis and, as a consequence, whether it represents a potential therapeutic target for inhibiting metastasis.

Although the involvement of IQGAP1 in several, metastasis-associated pathways make it a promising subject to study, this diverse array of interactions also complicates any investigation into specific mechanisms of action. IQGAP1 has several important functional domains, each of which binds to multiple proteins often implicated in metastasis. The calponin homology (CH) domain of IQGAP1 regulates cell migration by binding N-WASP and actin to promote actin assembly, branching and crosslinking at the leading edge^12,13^. The WW and IQ domains act as scaffolds for the Erk mitogen-activated protein kinase (MAPK) pathway, binding to B/C-Raf^14^, Mek 1/2^15^ and Erk 1/2^16^ to coordinate cell proliferation. IQGAP1’s RasGAP-related domain (GRD) binds to and stabilizes the active forms of the Rho GTPases Rac1 and Cdc42, which regulate cell migration and invasion^17^. IQGAP1’s RasGAP C-terminal (RGCT) domain associates with the exocyst complex proteins Sec3 and Sec8, and this association is necessary for the formation of mature invadopodia^18^, actin-rich protrusions used by tumor cells to degrade and invade through the extracellular matrix^19^. The RGCT also binds to both E-cadherin and beta-catenin, and this association weakens E-cadherin-mediated cell-cell adhesion^20^. Any or all of these interactions could be necessary for mediating potential pro-metastatic functions of IQGAP1, and the above proteins represent only a small subset of the total number of currently known IQGAP1 binding partners^21^. Still, understanding which domain or domains of IQGAP1 are critical for metastasis would be essential for the future design of therapeutics to disrupt these key interactions.

This study therefore seeks to answer directly the question of whether IQGAP1 is pro-metastatic and, if so, which of IQGAP1’s domains and functions are actually necessary for such an effect. We investigated the effects of IQGAP1 knockdown and knockout on both experimental and spontaneous metastasis models *in vivo*, and we found that reduction in IQGAP1 levels curtailed the formation of metastases from both melanoma and breast cancer cells. Notably, we found IQGAP1 knockout cells to be severely deficient in extravasation ability. This pro-metastatic phenotype seemed to be dependent on multiple domains of IQGAP1. These results firmly establish IQGAP1 as a metastasis promoter and suggest that it represents a promising candidate for future research, including as a potential therapeutic target.

## Results

### IQGAP1 knockdown reduces metastasis

**Figure 1:**
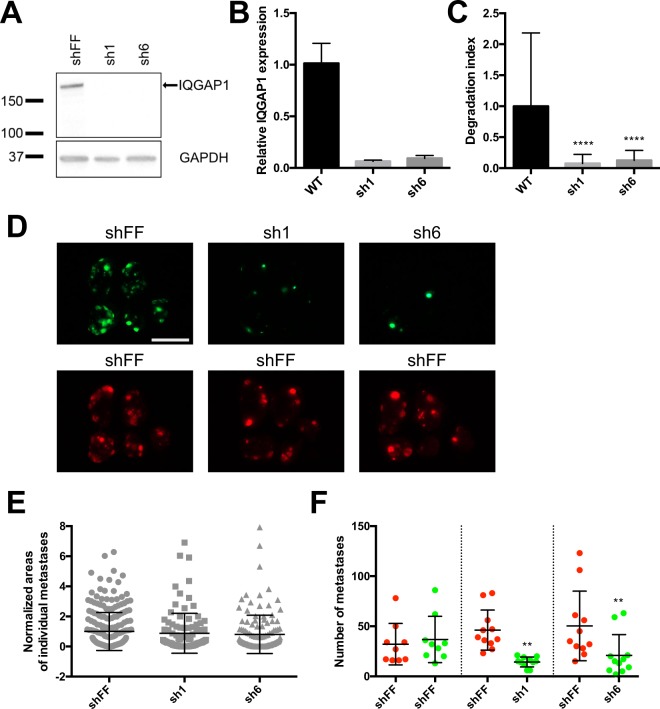
IQGAP1 knockdown reduces metastasis of MA2 cells from circulation. (**A**) Western blot of IQGAP1 and GAPDH in MA2 melanoma cells expressing shRNA against firefly luciferase (shFF) or IQGAP1 (sh1 and sh6). Molecular weight markers in kilodaltons (kDa) are indicated. Full-length IQGAP1 is shown with an arrow. Blot is cropped for clarity, with full-length blot presented in Supplementary Fig. S7A. (**B**) qPCR of expression of IQGAP1 relative to GAPDH in knockdown lines, normalized to WT parental MA2 cells. (**C**) Gelatin degradation assay, with area of gelatin degraded by IQGAP1 knockdown cells normalized to WT. (**D–F**) Tail-vein injection of a 50/50 mix of red (tdTomato) and green (ZsGreen) MA2 cells. n = 9–11 mice per group. (**D**) Representative images of lungs taken from mice 6 weeks after injection. Each red/green image pair is of the same set of lungs. Scale bar, 10 mm. (**E**) Size distribution of areas of individual green metastases from each experimental group. Values are normalized to red control tumor sizes from each mouse, as well as to green tumor sizes in the control group. (**F**) Number of metastases observed. Each red/green pair of cell lines (separated by dotted lines) was co-injected. ^**^P ≤ 0.01; ^****^P ≤ 0.0001; one-way ANOVA with Dunnett’s (**C**) or Šidák’s (**F**) multiple comparisons test.

To test the importance of IQGAP1 in metastasis *in vivo*, we first created stable IQGAP1 knockdowns in MA2 melanoma cells using miR30-based shRNAs (sh1 and sh6), along with control cells expressing shRNA against firefly luciferase (shFF) (Fig. 1A,B). Consistent with previous reports^22^, IQGAP1 knockdown caused cells to adopt a more spread and rounded morphology (Supplementary Fig. S1), along with a concordant reorganization of the actin and tubulin cytoskeletons (Supplementary Fig. S2). Furthermore, as previously reported^18^, IQGAP1 knockdown significantly reduced the degradation of gelatin by cells in an *in vitro* assay for invadopodial activity (Fig. 1C).

To test whether IQGAP1 knockdown has any effect on metastatic ability *in vivo*, 50/50 mixes of red fluorescent control cells with green fluorescent cells expressing either control or IQGAP1-targeting shRNA were then injected into the tail veins of immunocompromised mice to observe differences in lung metastasis (Fig. 1D). Individual metastases from IQGAP1 knockdown cells were not significantly different in size compared to control metastases (Fig. 1E), consistent with the fact that IQGAP1 knockdown had no effect on proliferation *in vitro* (Supplementary Fig. S3). However, IQGAP1 knockdown did reduce overall incidence of metastasis formation (Fig. 1F). Collectively, these results suggest that IQGAP1 expression is important in invasion and metastasis of tumor cells from circulation.

### IQGAP1 knockout reduces metastasis but not primary tumor growth

**Figure 2:**
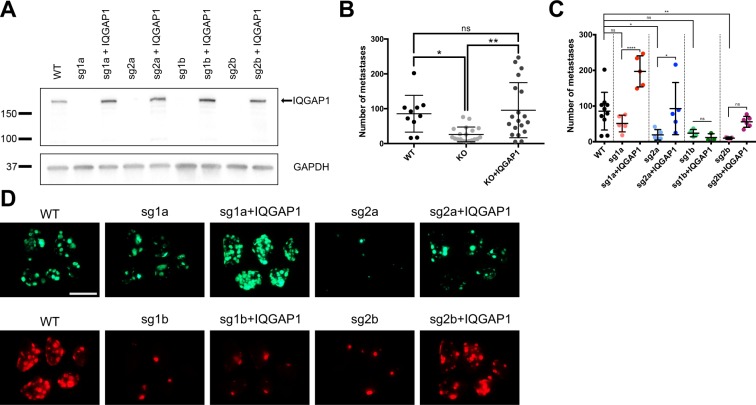
IQGAP1 knockout in MA2 cells reduces experimental metastasis *in vivo*. (**A**) Western blot of IQGAP1 and GAPDH in parental (WT), clonal IQGAP1 knockout (sg) and rescue (+IQGAP1) MA2 lines. Molecular weight markers in kDa are indicated. Full-length IQGAP1 is shown with an arrow. Blot is cropped for clarity, with full-length blot presented in Supplementary Fig. S7B. (**B**–**D**) Tail-vein injection of clonal IQGAP1 knockout and rescue lines, each injected individually. n = 10–19 mice per group. (**B**,**C**) Number of metastases observed, with all clonal knockout and rescue lines shown pooled (**B**) or separated by clonal line (**C**). (**D**) Representative images of lungs 6 weeks after injection. Scale bar, 10 mm. ^*^P ≤ 0.05; ^**^P ≤ 0.01; one-way ANOVA with Tukey’s multiple comparisons test.

To create a clean genetic background, we next generated complete IQGAP1 knockouts in MA2 cells using CRISPR-Cas9. Four clonal lines were established using two different sgRNAs against IQGAP1, and stable expression of wild-type IQGAP1 was then re-established in each knockout line as a rescue control (Fig. 2A), with rescue levels of IQGAP1 appearing somewhat higher compared to endogenous levels.

Each IQGAP1 knockout and rescue line was then assayed *in vivo* by tail-vein injection. Collectively, IQGAP1 knockout significantly reduced metastasis, while re-expressing wild-type IQGAP1 in the knockout lines restored metastatic ability (Fig. 2B), with individual results varying among the clonal knockout lines (Fig. 2C,D), consistent with the known variability observed to result from single-cell cloning in other cell lines^23^. These data broadly indicate that IQGAP1 knockout, like IQGAP1 knockdown, reduces metastasis of MA2 melanoma cells from circulation, whereas its overexpression increases metastasis.

**Figure 3:**
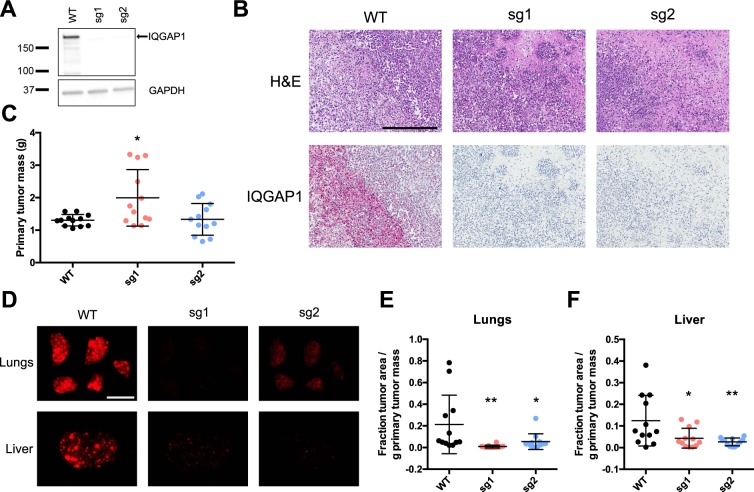
IQGAP1 knockout in LM2 breast cancer cells reduces spontaneous metastasis but not primary tumor growth. (**A**) Western blot of IQGAP1 and GAPDH in parental (WT) and clonal IQGAP1 knockout (sg) LM2 lines. Molecular weight markers in kDa are indicated. Full-length IQGAP1 is shown with an arrow. Blot is cropped for clarity, with full-length blot presented in Supplementary Fig. S7C. (**B**–**F**) Orthotopic mammary transplant of LM2 lines, with samples collected 8 weeks after injection. n = 12 mice per group. (**B**) Representative histology images of primary tumors showing H&E (top panels) or IQGAP1 (bottom panels) staining. Scale bar, 300 µm. (**C**) Distribution of primary tumor masses (g). (**D**) Representative images of lungs (top panels) and liver left lobes (bottom panels). Each pair of samples was taken from the same mouse. Scale bar, 10 mm. (**E**,**F**) Fraction of lung (**E**) or liver left lobe (**F**) surface area occupied by tumors, normalized to primary tumor mass (g) for each mouse. ^*^P ≤ 0.05; ^**^P ≤ 0.01; one-way ANOVA with Dunnett’s multiple comparisons test (all pairwise comparisons to WT).

To examine the relevance of IQGAP1 in metastasis in the context of a different cancer type, we also produced two clonal knockouts in LM2 breast cancer cells (an *in-vivo-*selected, highly metastatic derivative of MDA-MB-231)^24^, using the same two sgRNAs as above (Fig. 3A). These cells were then injected orthotopically into the mammary fat pads of immunocompromised mice to allow formation of primary tumors and ensuing metastasis. IQGAP1 knockout was total and was observed throughout the primary tumors *in vivo*, in contrast with wild-type tumors, which had extensive IQGAP1 expression (Fig. 3B and Supplementary Fig. S4).

However, despite the complete lack of IQGAP1, there was no reduction in primary tumor growth; in fact, one of the clonal knockout lines had significantly increased primary tumor mass (Fig. 3C). We compared metastatic burden in the lungs and liver between the wild-type and IQGAP1 knockout cell lines (Fig. 3D) and observed an overall reduction in metastasis to both organs following the loss of IQGAP1 (Fig. 3E,F), implying that IQGAP1 has an effect on metastasis independent of primary tumor growth. IQGAP1 knockout thus reduced metastasis *in vivo* in both melanoma and breast cancer cell lines, indicating a more generally conserved role for IQGAP1 in the metastatic cascade.

### IQGAP1 knockout reduces extravasation

Metastasis from circulation, despite comprising only the latter half of the metastatic cascade, still requires a number of distinct steps to be completed before circulating tumor cells can grow into overt metastases, including arrest, extravasation, early survival and eventual outgrowth^25^. Of these steps, the importance of IQGAP1 in invadopodial activity (Fig. 1C) is of particular relevance to extravasation, since extravasation of cells from circulation has been shown to require invadopodia^26^. We therefore hypothesized that IQGAP1 may be exerting its pro-metastatic effects during extravasation.

**Figure 4:**
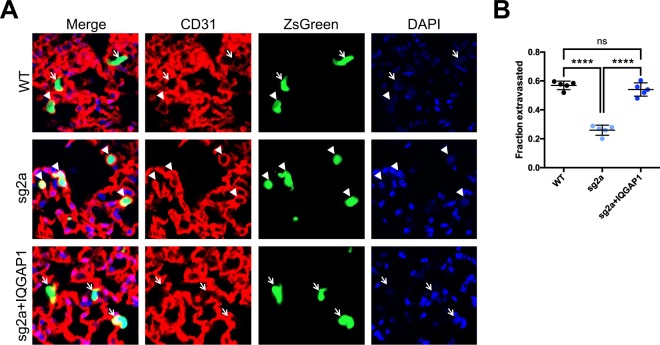
IQGAP1 knockout reduces extravasation of MA2 cells *in vivo*. (**A**) Representative images of parental, IQGAP1 knockout or rescued cells (ZsGreen), vasculature (CD31), and nuclei (DAPI) 20 hours after tail-vein injection. Extravasated cells (arrows) and cells still in vasculature (arrowheads) are indicated. Scale bar, 10 µm. (**B**) Fraction of observed cells that had extravasated. n = 5 mice per group. ns, P > 0.05; ^****^P ≤ 0.0001; one-way ANOVA with Tukey’s multiple comparisons test.

To evaluate more directly at which stage of the metastatic cascade IQGAP1 expression may be crucial, we performed an *in vivo* extravasation assay by injecting MA2 cells into mouse tail veins, then collected lungs after 20 hours. By comparing ZsGreen cell staining and CD31 vascular staining using confocal microscopy, we quantified whether each cell remained entirely within the vasculature (non-extravasated) or had emerged (extravasated), and we calculated what fraction of tumor cells had extravasated by this time (Fig. 4A). For this and future experiments, we selected for further study one clonal knockout line (sg2a) whose knockout and rescued cellular phenotypes most closely matched those of IQGAP1 knockdown and wild-type cells, respectively. Compared with wild-type cells, the extravasation rate of IQGAP1 knockout cells was halved, while re-expression of wild-type IQGAP1 in the same knockout background completely restored extravasation ability (Fig. 4B). These results suggest that IQGAP1 expression is crucial for extravasation of MA2 cells, and that IQGAP1’s effects on extravasation may account for much of its pro-metastatic ability in this system.

### IQGAP1’s effects on metastasis require multiple domains

**Figure 5:**
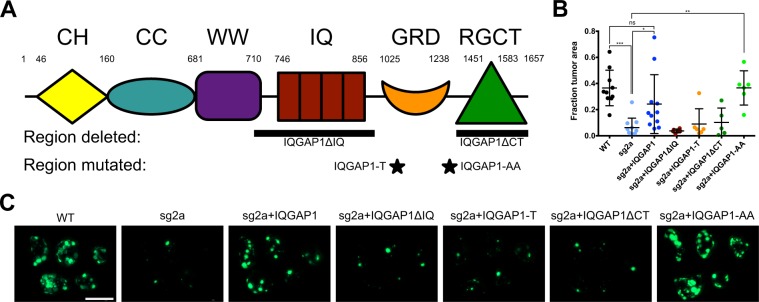
Effects of mutant forms of IQGAP1 on experimental metastasis in MA2 cells. (**A**) Domain structure of human IQGAP1, including calponin homology (CH), coiled coil (CC), WW, IQ, GAP-related domain (GRD) and RasGAP C-terminal (RGCT) domains, adapted from Brown and Sacks^27^. Shown below are regions of IQGAP1 deleted or mutated in the IQGAP1 mutant constructs used in this study: IQGAP1ΔIQ, IQGAP1-T1050AX2 (IQGAP1-T), IQGAP1-S1441A/S1443A (IQGAP1-AA) and IQGAPΔCT. (**B**,**C**) Tail-vein injection of parental (WT) or clonal IQGAP1 knockout line sg2a, rescued with either WT or mutant IQGAP1. n = 5–11 mice per group. (**B**) Fraction of lung surface area occupied by tumors. (**C**) Representative images of lungs 6 weeks after injection. Scale bar, 10 mm. ns, ^*^P ≤ 0.05; ^**^P ≤ 0.01; ^***^P ≤ 0.001; one-way ANOVA with Šidák’s multiple comparisons test.

Finally, we sought to explore which domains and functions of IQGAP1 may be necessary for its effects on metastasis. In addition to its eponymous IQ motifs and RasGAP-related domain (GRD), IQGAP1 contains calponin homology (CH), coiled-coil (CC), WW and RasGAP C-terminal (RGCT) domains (Fig. 5A)^27^. Of particular note, the IQ domain of IQGAP1 binds to the MAPK proteins B/C-Raf and Mek1/2^27^, as well as the metastasis promoter YAP^28^; the GRD binds to the Rho GTPases Rac1 and Cdc42^27^; and the RGCT binds to the exocyst complex proteins Sec3/8^18^. Any of these domains and binding interactions could plausibly be required for IQGAP1 to promote metastasis.

Consequently, we made MA2 cell lines expressing various mutant forms of IQGAP1 in the background of one of our clonal knockouts (sg2a): one mutant lacking all four IQ motifs (IQGAP1ΔIQ), another unable to bind Rac1 and Cdc42 (IQGAP1-T)^29^, a mutant lacking two major phosphorylation sites (IQGAP1-AA) and a final mutant missing the entire RasGAP C-terminal domain (IQGAPΔCT) (Fig. 5A and Supplementary Fig. S5). When we observed these cells in culture, we noted that IQGAP1-T and IQGAP1-AA were able to revert knockout cells from a more spread morphology towards the elongated, wild-type MA2 cell shape, while IQGAP1ΔIQ and IQGAPΔCT had little effect on apparent cell morphology (Supplementary Fig. S6).

Cell lines expressing these IQGAP1 mutants were then injected into mouse tail veins to assay for differences in lung metastasis (Fig. 5B,C). Consistent with our earlier results, we observed that IQGAP1 knockout reduced metastasis compared to wild-type cells, while re-expression of wild-type IQGAP1 rescued metastasis formation. However, expression of most of the IQGAP1 mutants (IQGAP1ΔIQ, IQGAP1-T and IQGAPΔCT) was unable to rescue metastatic ability. Only cells expressing IQGAP1-AA had metastasis formation restored to the level of wild-type cells. These results indicate that multiple domains of IQGAP1 are required for metastasis.

## Discussion

IQGAP1 is involved in a myriad of pathways commonly associated with cancer and metastasis, including proliferation, migration and invasion. However, despite this accumulation of circumstantial evidence, direct proof of IQGAP1’s importance in metastasis *in vivo* has been lacking. Furthermore, the diversity of roles performed by IQGAP1 could conceivably implicate it in any or all of the steps of the metastatic cascade: invasion from the primary tumor, intravasation, survival in circulation, extravasation, and outgrowth of the metastatic tumor. Lastly, given its enormous number of binding partners (well over a hundred)^21^ and functions, it is unclear exactly which of them, either individually or in combination, would be necessary for mediating any pro-metastatic effects. These broad uncertainties represent a diverse array of challenges in understanding the behavior and potential significance of IQGAP1 in metastasis.

In this study, we sought to begin the process of unraveling the complicated web of IQGAP1’s interrelated functions as they relate to metastasis through the use of direct, *in vivo* experiments. Reduction in IQGAP1 expression, through both knockdown and knockout, reduced experimental metastasis of MA2 melanoma cells from circulation by two- to three-fold on average (Figs. 1F and 2B), while re-expression of IQGAP1 was generally able to rescue this metastatic ability, though it is possible that increased levels of IQGAP1 compared to endogenous could have contributed to this rescue. Furthermore, IQGAP1 knockout reduced spontaneous metastasis of LM2 breast cancer cells from primary tumors to both the lungs and liver by a similar or even greater degree (Fig. 3E,F), even when normalizing for any changes in primary tumor mass (Fig. 3C). These results clearly show that IQGAP1 expression is important for metastasis in these systems, and the consistency of this effect in both melanoma and breast cancer models implies that IQGAP1 may be broadly relevant in metastasis across multiple cancers.

If IQGAP1 is, indeed, regulating metastasis, then at which stage or stages of the metastatic cascade is it having an effect? Given that IQGAP1 knockdown and knockout both decrease experimental metastasis from circulation, IQGAP1 must at least be relevant in the later stages of the metastatic cascade, including arrest, extravasation, early survival and proliferation into a full metastatic tumor. Individual IQGAP1 knockdown metastases had a similar average size compared to wild-type metastases (Fig. 1E), suggesting that IQGAP1 is not having a marked effect on metastatic proliferation, consistent with *in vitro* data (Supplementary Fig. S3), or else we would expect that IQGAP1 knockdown metastases would be significantly smaller. Instead, there is simply a diminished number of metastases forming. However, given IQGAP1’s observed effects on invadopodia activity, and the reported importance of invadopodia in extravasation^26^, we hypothesized that IQGAP1 might be involved in metastasis at the extravasation step. We observed a two- to three-fold decline in extravasation *in vivo* of IQGAP1 knockout cells compared to either wild-type or IQGAP1 rescue cells (Fig. 4B). The magnitude of this change is comparable to that of the overall decrease in metastasis due to IQGAP1 knockout (Fig. 2B), suggesting that much, if not most, of the observed difference in metastasis is likely due to differential extravasation ability. These data do not rule out the possibility that IQGAP1 may also have a lesser but still substantial effect on arrest in circulation and early survival, particularly given its known regulation of cell-cell adhesion and MAPK signaling^14–16,30^. Furthermore, the fact that IQGAP1 knockout reduces spontaneous metastasis from primary tumors (Fig. 3E,F) at least as much, if not more, than it reduces metastasis from circulation (Fig. 2B) may mean that IQGAP1 has some additional influence in the early stages of the metastatic cascade. Given IQGAP1’s effects on invadopodia (Fig. 1C) and extravasation (Fig. 4), this influence is most likely to apply during invasion of cancer cells from a primary tumor and subsequent intravasation into circulation. This is further supported by previous research demonstrating that IQGAP1 is overexpressed at the invasive front of human colorectal carcinomas^31^, and that breast cancer cells overexpressing IQGAP1 form more invasive tumors in mice^5^. Additional, detailed studies will be required to isolate more specifically all the individual steps of the metastatic cascade in which IQGAP1 expression is relevant.

IQGAP1 may also promote tumor progression. In this study, we primarily reduced IQGAP1 expression in cell lines that were already highly proliferative and metastatic, and we did not see any reduction in primary tumor growth from LM2 breast cancer cells (Fig. 3C). However, previous results in the MCF-7 breast cancer cell line showed that altering IQGAP1 expression could affect primary tumor growth^5^. These results could be due to differences in the cell lines used: the LM2 line is much more aggressive than MCF-7, and knocking out IQGAP1 may not have a large-enough effect to cause an observable difference in growth. Additionally, the process of single-cell cloning that we used to generate our knockout cell lines may naturally select for cells that are more proliferative. IQGAP1 knockdown also shifted cell morphology from a more stretched towards a more spread and rounded appearance (Supplementary Fig. S1), along with some cytoskeletal reorganization (Supplementary Fig. S2). These changes are consistent with morphological shifts observed in previous studies that demonstrated that changes in IQGAP1 expression can induce or reverse some forms of epithelial-mesenchymal transition (EMT)^8,22,32^. Moreover, IQGAP1 has been shown to bind to a number of known metastasis promoters, such as RhoC^33^ and YAP^28^, which already have potent effects of their own. Thus, in addition to the marked role for IQGAP1 in extravasation that we have identified, this versatile scaffold protein may have further roles in directing tumor growth, regulating EMT and coordinating other known metastasis promoters.

Finally, given IQGAP1’s enormous diversity of functions and binding partners, we attempted to identify which domains of IQGAP1 mediate its effects on metastasis by expressing several mutant forms of the protein in one of our clonal knockout MA2 lines (Fig. 5A, Supplementary Fig. S5). Only IQGAP1-AA, a mutant lacking two major phosphorylation sites^34^, was able to rescue metastasis to the level of wild-type cells, even slightly better than rescuing with wild-type IQGAP1 (Fig. 5B,C), indicating that phosphorylation at S1441 and S1443 is at least unnecessary for IQGAP1’s effects on metastasis and may even act in opposition. Interestingly, IQGAP1-AA was shown to be deficient in promoting neurite outgrowth in neuroblastoma cells compared to wild-type IQGAP1^34^. This suggests that the function of these phosphorylation sites is particular to specific functions of IQGAP1, rather than globally controlling binding, as with the Ca^2+^-dependent association of Calmodulin with IQGAP1, which negatively regulates IQGAP1’s other binding interactions^30,35,36^. Meanwhile, the remaining IQGAP1 mutants, those lacking all the IQ motifs (IQGAP1ΔIQ), those unable to bind Rac1 and Cdc42 (IQGAP1-T), or those missing the RasGAP C-terminal domain (IQGAPΔCT), were all unable to rescue metastasis in IQGAP1 knockout cells at all. The IQ motifs are responsible for binding the MAPK B/C-Raf^14^ and Mek1/2^15^, as well as EGFR^37^ and YAP^28^, involving this domain alone in cell survival, proliferation and migration. Indeed, the WW domain, which similarly binds the Erk1/2 MAPK, has previously been shown to be a valid therapeutic target: treatment with a peptide containing the IQGAP1 WW domain inhibited tumorigenesis and invasion of Ras-driven tumors in mice^6^. Moreover, YAP itself is a potent metastasis promoter^38^, so the interaction between IQGAP1 and YAP especially merits further investigation in the context of metastatic cancer. The inability of IQGAP1-T to regulate Rac1 and Cdc42 has very direct implications for cell migration and invasion, and cells expressing IQGAP1-T have been shown to be prone to form multiple leading edges^29^. It is therefore possible that the metastatic deficiency of cells expressing IQGAP1-T is due to their severely inhibited ability to extravasate out of blood vessels and invade into other tissues. However, the apparently low protein level of IQGAP1-T expressed in these cells (Supplementary Fig. S5) could mean that the inability of this mutant to rescue metastatic ability is due to insufficient expression, although the ability of even low levels of IQGAP1-T to alter cell morphology (Supplementary Fig. S6) indicates that its expression is having some effect. The RGCT domain, much like the IQ domain, binds to a diverse array of proteins, including beta-catenin, E-cadherin, CLIP-170, APC and Sec3/8^39^. Thus, this domain is also critical in regulating proliferation, cell-cell adhesion, migration and invasion. This last function may be the most relevant in our study. The association between the exocyst complex proteins Sec3/8 and IQGAP1’s RGCT domain is known to be crucial for the formation of mature invadopodia^18^, which are involved in both intravasation and extravasation^26,40^. Consequently, it is probable that the RGCT is directly involved in mediating IQGAP1’s effects on extravasation (Fig. 4). Together, the failure of the individual IQGAP1ΔIQ, IQGAP1-T and IQGAPΔCT mutants to rescue metastasis to any significant degree implies that IQGAP1’s effects in metastasis require several of its domains and functions, or at least a combination of binding partners that associate with different regions of the protein. More targeted studies, such as by blocking binding of specific proteins to IQGAP1, would be useful in isolating the particular binding partners important for metastasis.

In this study, we therefore present clear evidence that IQGAP1 is critical for metastasis *in vivo*, both in melanoma and breast cancer models. This need for IQGAP1 is due largely to effects on extravasation, though other steps of the metastatic cascade may also be influenced by IQGAP1. These effects on metastasis require multiple domains of IQGAP1, involving at least several of the numerous binding partners of this versatile scaffold protein. The exact roles of IQGAP1 in metastasis merit further study, and IQGAP1 may represent a therapeutic target for metastatic cancer.

## Methods

### Cell lines

The human melanoma MA2 cell line^41^ and the human mammary carcinoma LM2-mCherry cell line (originally developed in the lab of Joan Massagué^24^, a kind gift from Daniel Haber) were both cultured in HyClone high-glucose DMEM (ThermoFisher) supplemented with 2 mM glutamine and 10% fetal bovine serum (FBS, Invitrogen) in a 37 °C incubator with 5% CO_2_. Phase-contrast images of cells in culture were taken with a Zeiss Axiovert 200 microscope using a 10× or 5× objective.

### Expression vectors

All IQGAP1 constructs were cloned into the pHAGE-IRES-puro vector (a kind gift from David Benjamin; the original pHAGE vector backbone itself was a kind gift from Tyler Jacks). pHAGE-IQGAP1-IRES-Puro (wild-type IQGAP1) was cloned from IQGAP1 cDNA (GE Healthcare, accession BC139731). The pcDNA3-myc-IQGAP1-ΔIQ^42^ and pcDNA3-myc-IQGAP1-S1441A/S1443A^34^ vectors (referred to as IQGAP1ΔIQ and IQGAP1-AA in this study) were kind gifts from David Sacks. The pEGFP-IQGAP1-T1050AX2 (referred to in this study as IQGAP1-T, originally developed in the lab of Kozo Kaibuchi^29^) and pEGFP-IQGAP1-T1050AX2-ΔCC + RGC^18^ vectors were kind gifts from Philippe Chavrier. The T1050AX2 mutation was removed from IQGAP1-T1050AX2-ΔCC + RGC by cloning in the same region from wild-type IQGAP1, and the resulting construct is referred to as IQGAP1ΔCT in this study. MA2 cells were made green or red fluorescent through expression of pHAGE-ZsGreen-IRES-Hygro or pHAGE-TdTomato-IRES-Hygro, respectively (kind gifts from David Benjamin). Vectors for IQGAP1 knockdown and knockout are described in the section below. Retroviral and lentiviral production and transduction of cells was performed as previously described^43^.

### Gene knockdown and knockout

miR30-based shRNAs targeting IQGAP1 for knockdown were designed using a tool developed by the lab of Michael Hemann (shrna.mit.edu) and cloned into MSCV-Puro-miR30, as previously described^38,43^. An shRNA against Firefly luciferase (shFF) was used as a control. sgRNAs for IQGAP1 knockout were designed using a tool developed by the lab of Michael Boutros (e-crisp.org) and cloned into lentiCRISPRv2^44,45^. MA2 and LM2 cells were transiently transfected with lentiCRISPRv2 containing one of two sgRNAs against IQGAP1 (sg1 or sg2), then selected with puromycin. Cell populations were then sub-cloned to single cells. Following expansion, clonal cell lines were tested for IQGAP1 expression by western blotting (see next section). In this study, two clonal MA2 lines (a and b) were generated with each sgRNA (sg1 and sg2), resulting in four different clonal knockout lines (sg1a, sg1b, sg2a and sg2b). Each sgRNA was also used to generate a clonal LM2 line, resulting in two clonal knockout lines (sg1 and sg2). qRT-PCR analyses of all three IQGAPs in the knockout (MA2 and LM2) and rescue (LM2) lines showed insignificant levels of mRNA for IQGAP2 in any of these lines, in concordance with data from the human protein atlas (https://www.proteinatlas.org/ENSG00000145703-IQGAP2/tissue) showing very low levels in skin and breast. Positive controls with RNA extract from human liver tissue confirmed the primers used. There were no significant changes in mRNA levels for IQGAP3, and IQGAP1 mRNA levels were as expected according to knockdown, knockout and rescue status (data not shown). Thus, these cell lines do not appear to be compensating for a lack of IQGAP1 by significantly upregulating either IQGAP2 or IQGAP3.

### Immunoblotting, immunohistochemistry and quantitative PCR

For immunoblotting, cells were lysed in Cell Lysis Buffer (Cell Signaling Technology) containing a complete mini-protease inhibitor cocktail (Roche) and a phosphatase-inhibitor cocktail (PhosSTOP, Roche), then 10 µg of protein lysate were separated by SDS-PAGE using 4–20% gradient gels (Bio-Rad), transferred to nitrocellulose membranes, assayed by immunoblotting, and imaged with a Tanon 5200CE Chemi-Image System (Tanon). Primary antibodies were used at the following dilutions: rabbit anti-IQGAP1, 1:1000 (ab133490, Abcam); and mouse anti-GAPDH, 1:5000 (MAB374, Millipore). Immunohistochemical staining of IQGAP1, as well as hematoxylin and eosin staining, were performed as previously described, with rabbit anti-IQGAP1 used at a 1:500 dilution (sc-10792, Santa Cruz)^46^.

For quantitative PCR (qPCR), cells were lysed in TRIzol (Invitrogen), RNA was isolated according to the manufacturer’s instructions, and cDNA was synthesized by reverse transcription using the First-Strand cDNA Synthesis Kit (Promega). qPCR reactions were performed using SYBR Green Supermix (Bio-Rad) according to the manufacturer’s instructions, and data analysis was performed using Bio-Rad CFX Manager Software. PCR conditions were 95 °C for 10 min, followed by 40 cycles of 95 °C for 20 s, 60 °C for 30 s, and 72 °C for 30 s. The following primers were used: IQGAP1-forward, TTCTATGCAGCTTTCTCGGG; IQGAP1-reverse, CTGTCGAACTAAGTATCCACGG; IQGAP2-forward, GCTAGGGGAAATCGGCGAG; IQGAP2-reverse, TGCAGAGAGCCTTTCATCGT; IQGAP3-forward, GGCTGGGCAGCCTATGAAC; IQGAP3-reverse, CATTCCGAAGGCTCTCCTCC; GAPDH-forward, ACATCGCTCAGACACCATG; GAPDH-reverse, TGTAGTTGAGGTCAATGAAGGG.

### Immunofluorescence and gelatin degradation assay

Immunofluorescence staining was performed as described elsewhere^47^, except that normal goat serum was used to block for goat secondary antibodies. TRITC-phalloidin (1:1000, Sigma) was used to detect actin, along with rabbit anti-α-tubulin (1:500, ab18251, Abcam). To label cover slips with fluorescent gelatin, 18 mm circular No. 2 cover glass (VWR) was washed with a 2:1 mixture of nitric to hydrochloric acid for 2 hr, rinsed with 70% ethanol, then coated with 50 µg/ml poly-D-lysine for 20 min and fixed with 0.5% glutaraldehyde for 15 min. After washing with PBS, cover slips were then coated with FITC-gelatin (Molecular Probes) mixed with 2% sucrose. Cover slips were then coated with 20 µg/ml fibronectin (Advanced BioMatrix) and quenched with 5 mg/ml sodium borohydride (Sigma). 30,000 cells were added to each gelatin-coated cover slip. Cells were fixed 5 hours after plating with 4% paraformaldehyde and stained with 0.5 µg/ml DAPI (ThermoFisher). All images were taken with a Zeiss Axiovert 200 microscope using a 63× objective. Quantification of gelatin degradation area was conducted with ImageJ, and at least 50 cells were counted per line.

### Proliferation assay

Cell proliferation was measured with the Incucyte Zoom System (Essen Bioscience). 10,000 cells were seeded in 10 replicate wells of a 96-well plate per cell line. Phase-contrast images were captured every 3 hours over 160 hours total to calculate percent confluence. 4 images were quantified per well per time point.

### Tumor growth and metastasis assays

For tail-vein end-stage metastasis assays (excluding the extravasation assay detailed below), 1 × 10^6^ MA2 cells were injected into the lateral tail veins of 6–10-week-old male NOD-SCID mice (Jackson Laboratory) in 100 μL of Hanks’ Balanced Salt Solution (HBSS, Gibco). For mixing experiments (Fig. 1C–E), the cells injected were a 50/50 mix of TdTomato-labeled shFF control cells and ZsGreen-labeled experimental cells (either shFF or IQGAP1-targeting sh1 or sh6). 6 weeks after injection, lung lobes were dissected and then imaged with a Leica M165 FC dissecting microscope. ZsGreen- or TdTomato-labeled metastases were counted visually, and tumor burden was quantified by dividing tumor area by total tissue area using ImageJ. For orthotopic mammary transplant assays, 1 × 10^5^ LM2 cells were injected into the #4 mammary fat pads of 6- to 10-week old female NOD/SCID/IL2Rγ-null mice (Jackson Laboratory) in 25 μL of HBSS. 8 weeks after injection, primary tumors were dissected, weighed and fixed in 3.8% formaldehyde for subsequent paraffin embedding and sectioning. Lungs and liver left lobes were also collected and imaged as described above for tail-vein metastasis assays. All procedures were performed according to the animal protocol approved by MIT’s Department of Comparative Medicine and Committee on Animal Care.

### *In vivo* extravasation assay

One hundred microliters of cell suspension in PBS (1 × 10^6^ cells) were injected via lateral tail vein of NOD/SCID/gamma mice. Mice were euthanized, and lungs were collected after inflation with 4% formaldehyde and 0.3% Triton X-100 at 20 hours post injection. Fixed lungs were then cut to thin slices (0.5 mm thick) by scalpel and stained with anti-CD31 (5533070, BD Biosciences, 1:100) and Alexa 594-conjugated goat anti-rat IgG (Molecular probes). Images were taken with a Nikon A1R laser scanning confocal microscope using a 40× objective.

### Statistical analysis

All data were expressed as mean ± standard deviation. Statistical analysis was conducted with GraphPad Prism 6 (GraphPad Software). All comparisons were made using one-way ANOVA, with Dunnett’s, Tukey’s or Šidák’s multiple comparisons test as appropriate, with P ≤ 0.05 considered significant. Significance levels were indicated as follows: ns, P > 0.05; ^*^P ≤ 0.05; ^**^P ≤ 0.01; ^***^P ≤ 0.001; ^****^P ≤ 0.0001.

## Supplementary information

Supplementary information

## Data Availability

All data generated or analyzed during this study are included in this article and its Supplementary Material.

## References

[1] Gupta GP, Massagué J (2006). Cancer Metastasis: Building a Framework. Cell.

[2] Siegel RL, Miller KD, Jemal A (2018). Cancer statistics, 2018. CA. Cancer J. Clin..

[3] Steeg PS (2016). Targeting metastasis. Nat. Rev. Cancer.

[4] White CD, Brown MD, Sacks DB (2009). IQGAPs in cancer: A family of scaffold proteins underlying tumorigenesis. FEBS Lett..

[5] Jadeski L, Mataraza JM, Jeong H-W, Li Z, Sacks DB (2008). IQGAP1 Stimulates Proliferation and Enhances Tumorigenesis of Human Breast Epithelial Cells. J. Biol. Chem..

[6] Jameson KL (2013). IQGAP1 scaffold-kinase interaction blockade selectively targets RAS-MAP kinase–driven tumors. Nat. Med..

[7] Clark EA, Golub TR, Lander ES, Hynes RO (2000). Genomic analysis of metastasis reveals an essential role for RhoC. Nature.

[8] Wang X-X (2014). Targeted Knockdown of IQGAP1 Inhibits the Progression of Esophageal Squamous Cell Carcinoma *In Vitro* and *In Vivo*. PLoS ONE.

[9] Jin X (2015). The Overexpression of IQGAP1 and β-Catenin Is Associated with Tumor Progression in Hepatocellular Carcinoma *In Vitro* and *In Vivo*. PloS One.

[10] Diao B (2017). IQGAP1-siRNA inhibits proliferation and metastasis of U251 and U373 glioma cell lines. Mol. Med. Rep..

[11] Zhao H-Y (2017). IQ-domain GTPase-activating protein 1 promotes the malignant phenotype of invasive ductal breast carcinoma via canonical Wnt pathway. Tumor Biol..

[12] Bashour A-M, Fullerton AT, Hart MJ, Bloom GS (1997). IQGAP1, a Rac-and Cdc42-binding protein, directly binds and cross-links microfilaments. J. Cell Biol..

[13] Le Clainche C (2007). IQGAP1 Stimulates Actin Assembly through the N-Wasp-Arp2/3 Pathway. J. Biol. Chem..

[14] Ren J-G, Li Z, Sacks DB (2007). IQGAP1 modulates activation of B-Raf. Proc. Natl. Acad. Sci..

[15] Roy M, Li Z, Sacks DB (2005). IQGAP1 Is a Scaffold for Mitogen-Activated Protein Kinase Signaling. Mol. Cell. Biol..

[16] Roy M, Li Z, Sacks DB (2004). IQGAP1 Binds ERK2 and Modulates Its Activity. J. Biol. Chem..

[17] Hart MJ, Callow MG, Souza B, Polakis P (1996). IQGAP1, a calmodulin-binding protein with a rasGAP-related domain, is a potential effector for cdc42Hs. EMBO J..

[18] Sakurai-Yageta M (2008). The interaction of IQGAP1 with the exocyst complex is required for tumor cell invasion downstream of Cdc42 and RhoA. J. Cell Biol..

[19] Beaty BT, Condeelis J (2014). Digging a little deeper: The stages of invadopodium formation and maturation. Eur. J. Cell Biol..

[20] Kuroda S (1998). Role of IQGAP1, a Target of the Small GTPases Cdc42 and Rac1, in Regulation of E-Cadherin- Mediated Cell-Cell Adhesion. Science.

[21] Hedman Andrew C, Smith Jessica M, Sacks David B (2015). The biology of IQGAP proteins: beyond the cytoskeleton. EMBO reports.

[22] Dong, P. *et al*. Reactivation of epigenetically silenced miR-124 reverses the epithelial-to-mesenchymal transition and inhibits invasion in endometrial cancer cells via the direct repression of IQGAP1 expression. *Oncotarget*, 10.18632/oncotarget.7754 (2016). 10.18632/oncotarget.7754 PMC4991452 26934121

[23] Ben-David U (2018). Genetic and transcriptional evolution alters cancer cell line drug response. Nature.

[24] Minn AJ (2005). Genes that mediate breast cancer metastasis to lung. Nature.

[25] Lambert AW, Pattabiraman DR, Weinberg RA (2017). Emerging Biological Principles of Metastasis. Cell.

[26] Leong HS (2014). Invadopodia Are Required for Cancer Cell Extravasation and Are a Therapeutic Target for Metastasis. Cell Rep..

[27] Brown MD, Sacks DB (2006). IQGAP1 in cellular signaling: bridging the GAP. Trends Cell Biol..

[28] Sayedyahossein Samar, Li Zhigang, Hedman Andrew C., Morgan Chase J., Sacks David B. (2016). IQGAP1 Binds to Yes-associated Protein (YAP) and Modulates Its Transcriptional Activity. Journal of Biological Chemistry.

[29] Fukata M (2002). Rac1 and Cdc42 capture microtubules through IQGAP1 and CLIP-170. Cell.

[30] Li Z, Kim SH, Higgins JMG, Brenner MB, Sacks DB (1999). IQGAP1 and Calmodulin Modulate E-cadherin Function. J. Biol. Chem..

[31] Nabeshima K, Shimao Y, Inoue T, Koono M (2002). Immunohistochemical analysis of IQGAP1 expression in human colorectal carcinomas: its overexpression in carcinomas and association with invasion fronts. Cancer Lett..

[32] Su D, Liu Y, Song T (2017). Knockdown of IQGAP1 inhibits proliferation and epithelial-mesenchymal transition by Wnt/β-catenin pathway in thyroid cancer. OncoTargets Ther..

[33] Casteel DE (2012). Rho Isoform-specific Interaction with IQGAP1 Promotes Breast Cancer Cell Proliferation and Migration. J. Biol. Chem..

[34] Li Z (2005). IQGAP1 Promotes Neurite Outgrowth in a Phosphorylation-dependent Manner. J. Biol. Chem..

[35] Ho Y-D, Joyal JL, Li Z, Sacks DB (1999). IQGAP1 Integrates Ca2+/Calmodulin and Cdc42 Signaling. J. Biol. Chem..

[36] Joyal JL (1997). Calmodulin Modulates the Interaction between IQGAP1 and Cdc42 Identification of Iqgap1 By Nanoelectrospray Tandem Mass Spectrometry. J. Biol. Chem..

[37] McNulty DE, Li Z, White CD, Sacks DB, Annan RS (2011). MAPK Scaffold IQGAP1 Binds the EGF Receptor and Modulates Its Activation. J. Biol. Chem..

[38] Lamar JM (2012). The Hippo pathway target, YAP, promotes metastasis through its TEAD-interaction domain. Proc. Natl. Acad. Sci..

[39] White CD, Erdemir HH, Sacks DB (2012). IQGAP1 and its binding proteins control diverse biological functions. Cell. Signal..

[40] Gligorijevic B (2012). N-WASP-mediated invadopodium formation is involved in intravasation and lung metastasis of mammary tumors. J. Cell Sci..

[41] Xu L (2008). Gene Expression Changes in an Animal Melanoma Model Correlate with Aggressiveness of Human Melanoma Metastases. Mol. Cancer Res..

[42] Sokol SY, Li Z, Sacks DB (2001). The Effect of IQGAP1 on Xenopus Embryonic Ectoderm Requires Cdc42. J. Biol. Chem..

[43] Stern P (2008). A system for Cre-regulated RNA interference *in vivo*. Proc. Natl. Acad. Sci..

[44] Shalem O (2014). Genome-Scale CRISPR-Cas9 Knockout Screening in Human Cells. Science.

[45] Sanjana NE, Shalem O, Zhang F (2014). Improved vectors and genome-wide libraries for CRISPR screening. Nat. Methods.

[46] Rickelt Steffen, Hynes Richard O. (2018). Antibodies and methods for immunohistochemistry of extracellular matrix proteins. Matrix Biology.

[47] Krawczyk E, Suprynowicz FA, Sudarshan SR, Schlegel R (2010). Membrane Orientation of the Human Papillomavirus Type 16 E5 Oncoprotein. J. Virol..

