# Supplementary material for: The scaffold protein IQGAP1 is crucial for extravasation and metastasis
Source: Sci Rep. 2020 Feb 12;10:2439. doi: 10.1038/s41598-020-59438-w (PMC7015931; doi:10.1038/s41598-020-59438-w)
Supplement: Supplementary file 1 — Supplementary information [file 41598_2020_59438_MOESM1_ESM.docx]

**The scaffold protein IQGAP1 is crucial for extravasation and metastasis**

Jess D. Hebert,^1,2^ Chenxi Tian,^2^ John M. Lamar,^2,3^ Steffen Rickelt,^2^ Genevieve Abbruzzese,^2^ Xiaotie Liu^2^ and Richard O. Hynes*^1,2,4^

^1^ Department of Biology, Massachusetts Institute of Technology, Cambridge, Massachusetts 02139

^2^ Koch Institute for Integrated Cancer Research, Massachusetts Institute of Technology, Cambridge, Massachusetts 02139

^3^ Department of Molecular and Cellular Physiology, Albany Medical College, Albany, New York 12208

^4^ Howard Hughes Medical Institute, Chevy Chase, Maryland 20815

*Corresponding author:

Richard O. Hynes

Room 76-361, Koch Institute, MIT

77 Massachusetts Avenue, Cambridge, 02139

Phone: 617-253-6422

Fax: 617-253-8357

Email: [rohynes@mit.edu](mailto:rohynes@mit.edu)

**Supplementary Figures**

**Supplementary Figure 1. Observed morphology of MA2 cells in culture.**

Phase-contrast images of parental (WT) and IQGAP1 knockdown (sh1 and sh6) cells. Scale bar, 100 µm.

**Supplementary Figure 2. Cytoskeletal staining of parental and IQGAP1 knockdown MA2 cells.**

Immunofluorescence staining of actin (top panels), α-tubulin (middle panels) and DAPI (bottom panels) in parental (WT) and IQGAP1 knockdown (sh1 and sh6) cells. Scale bar, 10 µm.

**Supplementary Figure 3. *In vitro* proliferation assay of control and IQGAP1 knockdown MA2 cells.**

Observed percent confluence of control (shFF) and IQGAP1 knockdown (shIQGAP1-1 and shIQGAP1-6) MA2 cells in culture over the course of 160 hours. Each data point shown represents the average of 10 wells, with four images quantified per well for each timepoint.

**Supplementary Figure 4. Histological staining of LM2 primary tumors.**

Representative histology images of full primary tumors from parental (WT) or clonal IQGAP1 knockout (sg) lines, showing H&E (top panels) or IQGAP1 (bottom panels) immunohistochemical staining (red). Insets shown are the same as in Figure 3B. Boxes indicate areas from which insets were taken. Scale bars, 4 mm (full tumors) or 300 µm (insets).

**Supplementary Figure 5. Expression of IQGAP1 mutants.**

Western blot of IQGAP1 and GAPDH in clonal IQGAP1 knockout line sg2a, rescued with either WT or mutant IQGAP1. Molecular weight markers in kDa are indicated. Full-length IQGAP1 is shown with an arrow.

**Supplementary Figure 6. Observed morphology of IQGAP1 knockout and rescue cells in culture.**

Phase-contrast images of MA2 IQGAP1 clonal knockout line sg2a, and the same line rescued with WT (“+IQGAP1”) or mutant IQGAP1. Scale bar, 100 µm.

**Supplementary Figure 7. Full-length western blots.**

(A) Western blot of IQGAP1 and GAPDH in MA2 melanoma cells expressing shRNA against firefly luciferase (shFF) or IQGAP1 (sh1 and sh6). Full-length blot of Fig. 1A. (B) Western blot of IQGAP1 and GAPDH in parental (WT), clonal IQGAP1 knockout (sg) and rescue (+IQGAP1) MA2 lines. Full-length blot of Fig. 2A. (C) Western blot of IQGAP1 and GAPDH in parental (WT) and clonal IQGAP1 knockout (sg) LM2 lines. Full-length blot of Fig. 3A. For all blots, molecular weight markers in kDa are indicated, and full-length IQGAP1 is shown with an arrow.
